# Soil Methane Sink Capacity Response to a Long-Term Wildfire Chronosequence in Northern Sweden

**DOI:** 10.1371/journal.pone.0129892

**Published:** 2015-09-15

**Authors:** Niall P. McNamara, Ruth Gregg, Simon Oakley, Andy Stott, Md. Tanvir Rahman, J. Colin Murrell, David A. Wardle, Richard D. Bardgett, Nick J. Ostle

**Affiliations:** 1 Centre for Ecology & Hydrology, Lancaster Environment Centre, Lancaster, United Kingdom; 2 NERC Life Sciences Stable Isotope Facility, Centre for Ecology & Hydrology, Lancaster Environment Centre, Lancaster, United Kingdom; 3 Department of Microbiology and Hygiene, Bangladesh Agricultural University, Mymensingh, Bangladesh; 4 School of Environmental Science, University of East Anglia, Norwich Research Park, Norwich, United Kingdom; 5 Department of Forest Ecology and Management, Swedish University of Agricultural Sciences, Umeå, Sweden; 6 Faculty of Life Sciences, Michael Smith Building, The University of Manchester, Oxford Road, Manchester, United Kingdom; 7 Lancaster Environment Centre, Lancaster University, Lancaster, United Kingdom; USDA-ARS, UNITED STATES

## Abstract

Boreal forests occupy nearly one fifth of the terrestrial land surface and are recognised as globally important regulators of carbon (C) cycling and greenhouse gas emissions. Carbon sequestration processes in these forests include assimilation of CO_2_ into biomass and subsequently into soil organic matter, and soil microbial oxidation of methane (CH_4_). In this study we explored how ecosystem retrogression, which drives vegetation change, regulates the important process of soil CH_4_ oxidation in boreal forests. We measured soil CH_4_ oxidation processes on a group of 30 forested islands in northern Sweden differing greatly in fire history, and collectively representing a retrogressive chronosequence, spanning 5000 years. Across these islands the build-up of soil organic matter was observed to increase with time since fire disturbance, with a significant correlation between greater humus depth and increased net soil CH_4_ oxidation rates. We suggest that this increase in net CH_4_ oxidation rates, in the absence of disturbance, results as deeper humus stores accumulate and provide niches for methanotrophs to thrive. By using this gradient we have discovered important regulatory controls on the stability of soil CH_4_ oxidation processes that could not have not been explored through shorter-term experiments. Our findings indicate that in the absence of human interventions such as fire suppression, and with increased wildfire frequency, the globally important boreal CH_4_ sink could be diminished.

## Introduction

The Earth’s boreal forests lie between 45° and 70°N and account for one third of all forested lands [1], covering between 9–12 million km^2^. As such, they play a major role in global carbon (C) storage and greenhouse gas regulation [2]. It is estimated that 37% of all terrestrial C is stored in boreal forests, which is greater than combined temperate and tropical stocks [1]. Currently, there is mounting concern for these high latitude C stores as they are predicted to undergo some of the strongest warming resulting from global climate change [3]. A possible consequence of rising temperatures is enhanced likelihood of wildfire ignitions and conflagration of forest biomass and organic soil by lightning strikes and human activity. It has been suggested that wildfire events could increase in frequency, intensity and extent as a result of a greater availability of drier fuel materials following more persistent and extreme drought events, and longer growing seasons due to earlier snow melt [4]. To date, both increases and decreases in fire events have been recorded, with diminishing fire frequency being associated with improved fire suppression techniques and habitat fragmentation as a result of land-use change [4]. This means that climate-driven increases in severe wildfire events may in part be balanced by human activity to suppress forest fires. Nonetheless, boreal forest wildfires burn between 3 and 23.6 million ha/year, representing an average of 13 million ha/year [5]. Wildfire events can have significant, large scale and prolonged impacts on terrestrial C storage as a consequence of both direct and indirect effects. In the short term, fire results in the conversion of forest biomass and soil organic C to CO_2_ that is released to the atmosphere [6–7], whilst a smaller fraction of biomass enters soils as more recalcitrant black carbon [8]. Black carbon or charcoal additions can have varying effects on soil processes, at least for decades, including altering nutrient availability and increasing decomposition [9]. In the longer term, wildfires act as a fundamental regulator of succession, and thus C sequestration processes [2].

Globally, soils are the largest natural terrestrial CH_4_ sink through biological oxidation by methantrophic bacteria. It is widely accepted that in aerobic soils, methanotrophic activity [10] can be impaired for up to a century following soil disturbance events such as land use change or nitrogen addition [11]. To date, fire as a driver of this disturbance has received relatively little attention. At least in the short term (decadal), fire can increase [12–13], decrease [14] or have no change [15] for the net soil CH_4_ sink, however, the underlying processes are poorly understood and are likely to be ecosystem dependent. Beneath forested organic rich wet soils recent fires have also been shown to alter soils from CH_4_ sink to source [16]. Critically, however, the effects of increased or decreased wildfire frequency on soil CH_4_ processes over longer (centennial or millennial) time scales is uncertain. The paucity of detailed studies of microbial CH_4_ oxidation in boreal forest soils represents a serious gap in understanding that we sought to address in this study.

Here, we used a unique natural boreal forest fire chronosequence to determine the effects of historic fire disturbance on soil microbial methanogenesis and methanotrophy. This involved the study of 30 boreal forested islands located in freshwater lakes in Northern Sweden. These islands range in surface area from 0.02 to 15 ha. Lightning strikes were generally more frequent and thus more recent on larger islands; some large islands have burned as recently as 60 years ago while some small islands have not burned for 5,000 years [17–20]. Consistent with previous work across this island gradient, we have classified these islands as early (time since last fire (mean ± SE) = 585 ± 233 years; area > 10 ha; n = 10), mid (2,180 ± 385 years; area of 0.1 to 1.0 ha; n = 10) and late (3,250 ± 439 years; area < 0.1 ha; n = 10) successional stages [17–19]. Using this natural fire chronosequence and substituting space for time, we aimed to better understand how ecosystem retrogression, as driven by wildfires, affects the soil-atmosphere exchange of CH_4_. We made a series of in-situ and ex-situ measurements of soil CH_4_ fluxes to characterise net CH_4_ fluxes during two successive growing seasons across the gradient of 30 islands.

## Results and Discussion

**Fig 1:**
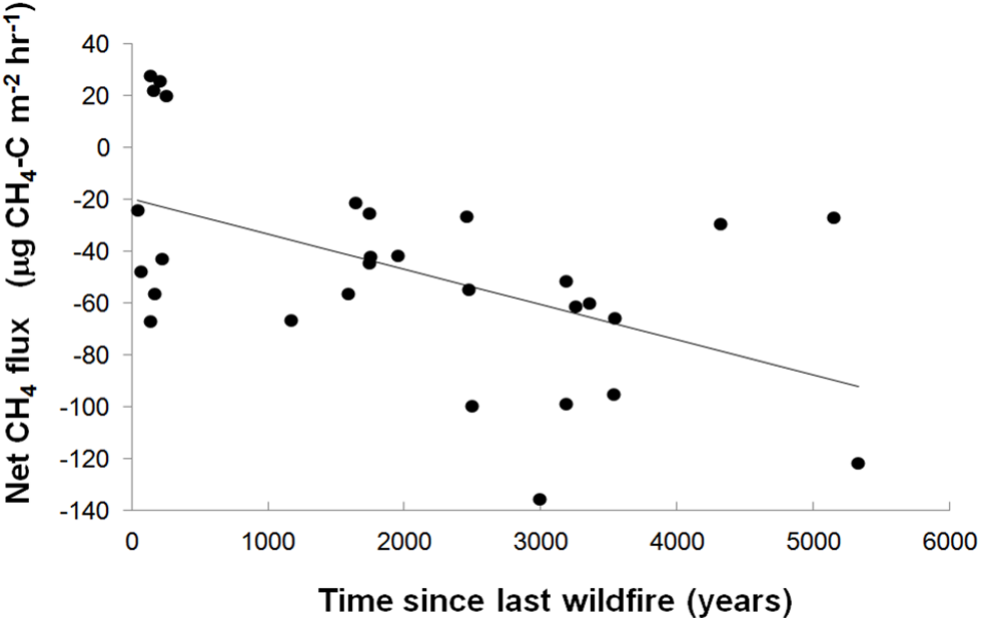
In situ CH_4_ flux versus time since last fire. *In situ* 2006 CH_4_ chamber flux data versus time since last fire (R^2^ = 0.4; P = 0.001). Each data point represents the mean of five measurements made on each island at the same time. 10 depth measurements were made per island.

**Fig 2:**
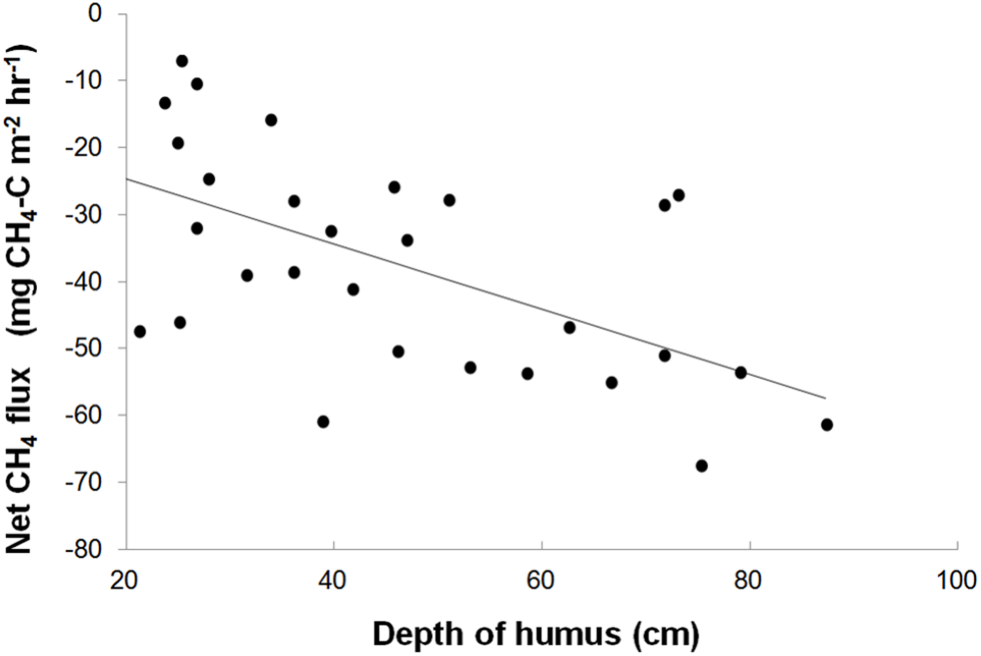
In situ CH_4_ flux versus depth of humus. *In situ* 2006 CH_4_ flux versus depth of humus (R^2^ = 0.35; P ≤ 0.001). Each data point represents the mean of five measurements made on each island at the same time. 10 depth measurements were made per island.

The effect of time elapsed since last wildfire event on *in situ* net soil CH_4_ fluxes was examined using a static chamber method (n = 5 per island, 30 islands). Results show that the net soil CH_4_ sink significantly increases (R^2^ = 0.40, P<0.001) with time since last wildfire disturbance (Fig 1). We propose that this effect is mediated by successional changes in the plant community since fire disturbance. As time since fire increases, vegetation community composition shifts from domination by early successional *Pinus sylvestris* and *Vaccinium myrtilus*, to the late successional *Picea abies* and *Empetrum hermaphroditum* [18, 20]. The late succession plant community is characterised by having recalcitrant litter inputs, impaired productivity and decomposer activity, and greater humus depth and C storage (S1 Table) [17–20]. The build-up of soil organic matter was observed to increase significantly with time since fire disturbance (R^2^ = 0.41; P ≤ 0.001), with a significant correlation between greater humus depth and net soil CH_4_ oxidation rates (R^2^ = 0.35; P ≤ 0.001; Fig 2). Soil moisture variation in the top 25 cm of humus, a common driver of short-term soil CH_4_ oxidation rates, was found to have a weak but positive correlation with the net CH_4_ oxidation rates (R^2^ = 0.15; P = 0.03, Table 1), whilst no relationship was identified with bulk density. Using island area classes that corresponded with plant successional classifications, early successional islands showed significantly lower net CH_4_ oxidation than islands at mid or late successional stages (Table 2). Overall, these results offer strong evidence that deeper soils of later post-fire successional forest stages provided conditions for enhanced methanotrophic activity.

**Table 1:** Soil moisture determinations (%) made from soil cores from early, mid and late succession islands.

Year	Depth (cm)	Early	Mid	Late	F	P
2006	0–25	54.2^a^ (3.7)	58.8^a,b^ (2.2)	66.6^b^ (1.7)	5.42	0.01
2007	0–20	71.3^a^ (4.8)	73.3^a^ (3.2)	74.5^a^ (0.9)	0.23	0.8
2007	20–40	66.9^a^ (13.3)	81.6^b^ (1.5)	76.8 ^ab^ (0.6)	3.45	0.05
2007	40–60	nd	78.3 ^a^ (0.8)	79.3 ^a^ (0.9)	0.39	0.55
2007	60–80	nd	nd	82.0 (2.7)	nd	nd

For 2006 all values in brackets are standard errors of the mean (n = 10). Values followed by the same letter in superscript indicate no significant difference at P < 0.05, d.f = 2,27. For 2007, 20–40 cm values in brackets are standard errors of the mean for early (n = 3), mid (n = 8) and late (n = 10), d.f = 2,18; 40–60 cm values in brackets are standard errors of the mean for mid (n = 3) and late (n = 8), d.f = 1,9; 60–80 cm values in brackets are standard errors of the mean for late (n = 5). ‘nd’ indicates where samples could not be obtained due to a shallow humus depth.

**Table 2:** Net CH_4_ oxidation rate of early, mid and late succession islands for *in situ* and *ex situ* soil core measurements.

Measurement	Early	Mid	Late	F	P
*In situ* 2006	-25.76^a^ (4.28)	-40.32^b^ (4.61)	-45.20^b^ (4.88)	4.84	0.02
*Ex situ* 2006 (25 cm core)	-11.08^a^ (2.31)	-12.81^a^ (2.50)	-9.09^a^ (2.82)	0.53	0.59
*Ex situ* 2007 (up to 85 cm core)	-6.38^a^ (2.55)	-11.99^a,b^ (2.44)	-16.52^b^ (2.90)	3.70	0.04

Values in brackets are standard errors of the mean (n = 10). Values followed by the same letter in superscript indicate no significant difference at P < 0.05, d.f = 2,27.

We targeted CH_4_ flux measurements on the uppermost 25 cm of the soil profile where previous studies have shown evidence of maximal methanotrophic activity [21–22]. Intact 25 cm deep soil cores (n = 4 per island x 30) were sampled from each island. These cores were then examined in *ex situ* laboratory incubations where we did not detect a relationship between net CH_4_ fluxes and time since last fire, or with successional stage (Table 2). This result, combined with net field CH_4_ flux data, suggests that significant CH_4_ oxidation must be occurring at depths greater than 25 cm. To investigate this we returned to the islands in of the following year and collected whole humus soil profile cores (up to 85 cm depth to bedrock) from each island. These were taken back to the laboratory to carry out CH_4_ flux measurements under controlled environmental conditions. Another possible cause for the lack of observed differences in CH_4_ oxidation rates from the 25 cm cores was our *ex-situ* methodologies; ex-situ approaches by their nature are disruptive, for example, through altering diffusional rates of CH_4_ from the atmosphere or by removing the supply of CH_4_ from deeper anaerobic regions. By measuring CH_4_ fluxes from deeper cores we aimed to confirm that this was not the case. During the second year’s campaign soils were considerably wetter than in the previous (Table 1). The observed rates of *ex situ* CH_4_ oxidation from the deep cores was lower than the previous *in situ* and *ex situ* measurements made during the previous year, however, net CH_4_ oxidation rates were in the order Late > Mid > Early successional islands (Table 2). Thus, comparing net CH_4_ oxidation results from our 25 cm and the deep cores indicate that methanotrophic activity is prevalent in deeper soil layers that form under the prolonged absence of wildfire.

**Fig 3:**
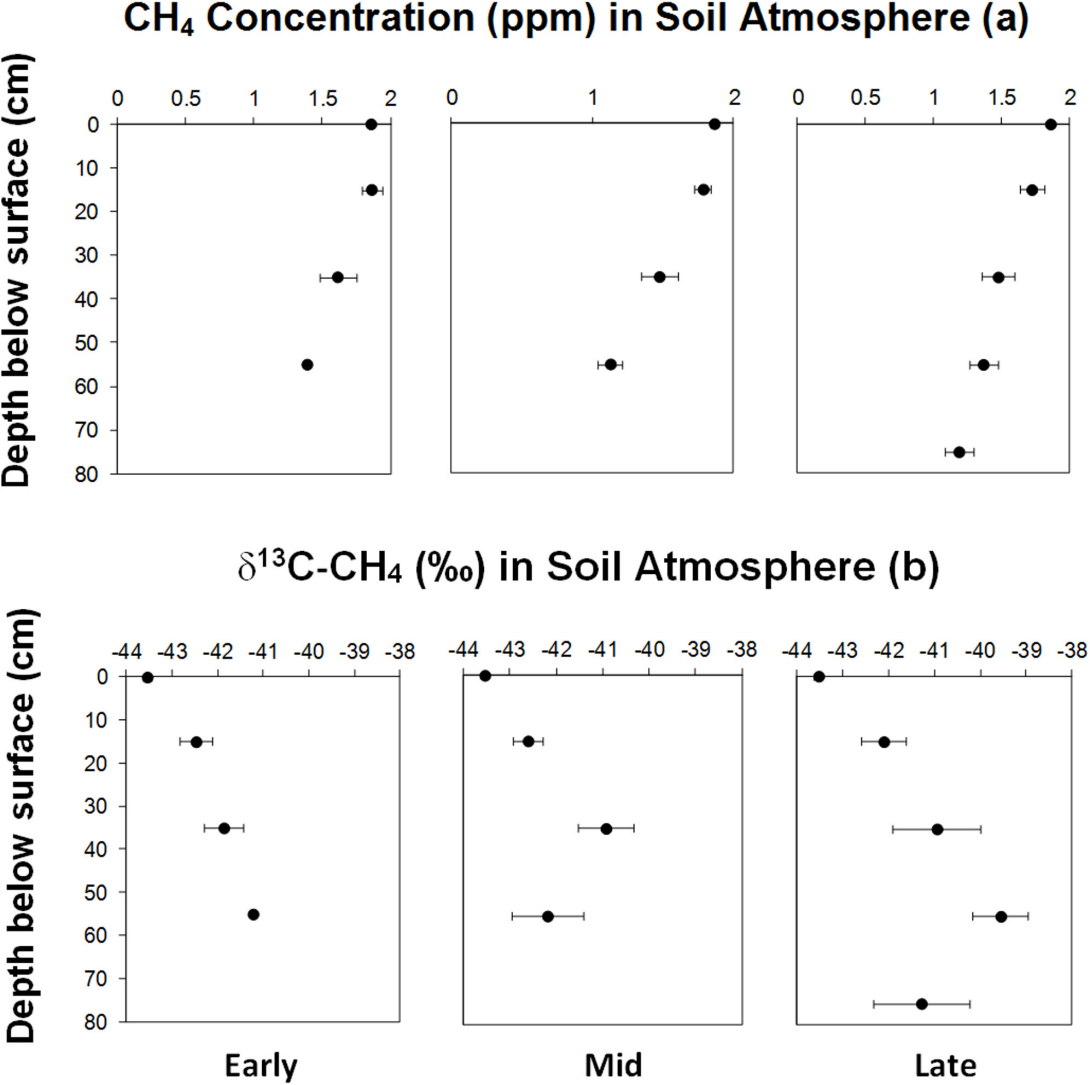
Mean *in situ* concentration depth profiles of CH_4_ (a) and distribution of δ^13^C-CH_4_ (b) in boreal forest soils. The depth at surface (0 cm) represents the ambient concentration and δ^13^C-CH_4_ value. Error bars represent ± standard error (n = 10). The changes in CH_4_ and CO_2_ concentration and δ^13^C-CH_4_ with depth were only significant in mid and late successional stages (P ≤ 0.05). Statistical analyses of data are shown in Table 2.

Based on our *in-situ* results from our first campaign we hypothesised that methanogenic processes were concurrent in deeper humus layers, thus potentially priming methanotrophic activity through provision of additional CH_4_ substrate supply [23]. During our second field campaign we therefore used a natural abundance stable isotope *in situ* approach to gain additional insight into CH_4_ processes at depth. For this, a series of sub-surface CH_4_ concentration measurements were made across the islands at a range of soil depths. These measurements allowed us to initially determine whether we could detect above ambient concentrations of CH_4_ as evidence of active methanogenesis in the soil profile. Concentration of CH_4_ measured in the humus profile were consistently below ambient levels, suggesting that either methanogenesis was not dominant in these soils during the peak growth season (Fig 3A), or that rapid oxidation of CH_4_ by the methanotroph community was removing any endogenous CH_4_ production. Analyses of the stable ^13/12^C isotope ratio of CH_4_ in these samples confirmed that methanogenesis was not occuring, as δ^13^C values of ^13^CH_4_ were enriched relative to atmospheric CH_4_ (Fig 3B). It has been shown that methanotrophs preferentially assimilate lighter isotopes, leaving residual CH_4_ that is ^13^C enriched [24]. Our results demonstrated that both CH_4_ concentrations and the δ^13^C values of ^13^CH_4_ were strongly influenced by the depth of the humus profile (Table 2). Using these data, we quantified the CH_4_ oxidation rate at sub-surface depths *in situ* using a ‘top-to-bottom difference’ ^13^C-CH_4_ modelling approach [24–25]. We found that soils typically oxidised between 2–5% of CH_4_ in the atmosphere (Table 3), with deeper soils leading to a higher cumulative CH_4_ oxidation rate (Table 4). As these soils were not found to produce CH_4_, the supply of this substrate to deeper soil profile was from the atmosphere. This increasing CH_4_ oxidation at depth corresponded to the sub-surface minimum CH_4_ concentrations detected in the humus profile recorded with our *in-situ* depth measurements (Fig 3A), and also in *ex situ* profile cores (up to 85 cm) taken during the second field campaign (S2 and S3 Tables). Collectively, these results support the notion that increasing time since wildfire has indirectly affected the CH_4_ oxidation process by enabling the development of deeper humus profiles which harbour the niches necessary for methanotrophs.

**Table 3:** Percentage of atmospheric CH_4_ oxidised in the soil profile and statistical analysis of whole profile % CH_4_ oxidised for early, mid and late succession islands.

	CH_4_ (%) oxidised at specific depth ranges									
Successional Stage	0–15 cm	n	0–35 cm	n	0–55 cm	n	0–75 cm	n	Whole profile	n
Early	1.47 (0.5)	10	2.30 (0.61)	6	3.18 (NA)	1	nd	0	2.02 (0.45)^a^	10
Mid	1.27 (0.45)	10	3.66 (0.85)	9	1.88 (0.9)	6	nd	0	3.1 (0.83)^a^	10
Late	1.96 (0.68)	10	3.73 (1.41)	10	5.63 (0.80)	9	3.14 (1.51)	4	5.09 (1.15)^a^	10

Model assumes transport of CH_4_ in to the soil by diffusion (α _transport_ = 1.0195). The whole profile estimate considers the total CH_4_ oxidised for the given depths of humus. Values in brackets are standard errors of the mean. For early and mid islands ‘nd’ indicates where samples could not be obtained due to a shallow humus depth. The number of depths sampled is given by n. For whole profile analyses values followed by the same letter in subscript indicate no significant difference at P < 0.05, F = 2.6129, P = 0.09176, d.f = 2, 27.

**Table 4:** Statistical analyses for the effects of, and interactions between, depth and sucessional stage on *in situ* profile CH_4_ concentration, δ^13^C-CH_4_ and % CH_4_ oxidised.

**CH**_**4**_ **concentration**	F	P
Stage (S)	3.2975	0.0529
Sample Depth (SD)	53.4777	0.0001
Stage x Sample Depth (S x SD)	2.5726	0.0883
δ^**13**^ **CH**_**4**_	F	P
Stage	2.061	0.1469
Sample Depth	8.336	0.0061
Stage x Sample Depth	0.150	0.8614
**% CH**_**4**_ **oxidised**	F	P
Stage	2.13987	0.1372
Sample Depth	8.03978	0.0070
Stage x Sample Depth	0.15000	0.8602

df: S = 2, 26, SD = 1, 42, S x SD = 2, 42

Our study reveals, for the first time, that across contrasting islands differing in exposure times to historical fire disturbance, net CH_4_ oxidation processes vary predictably. Specifically, we have shown that low productivity, old growth boreal forest ecosystems that have not burned for up to 5000 years develop deeper humus layers, leading to enhanced soil microbial CH_4_ oxidation. Crucially, our results indicate that disturbance of these humus C stores will threaten the stable microhabitat conditions required for methanotrophs to thrive. In the future, climate change is expected to result in increased wildfire ignitions from lightning and increased fire season length [26]. These same drivers are also expected to increase wildfire severity which will in turn promote soil organic matter loss and methanotrophic community disturbance. Together, these global changes could reduce the apparent natural CH_4_ sink capacity of late succession boreal forest soils, with potential feedback to greenhouse gas forced climate warming. Our results indicate that in the absence of human interventions, such as fire suppression, the globally important boreal CH_4_ sink could be diminished by future wildfire events.

## Materials and Methods

### Ethics Statement

This study was made on 30 freshwater islands in the Boreal forest region of northern Sweden. These islands are part of an archipelago of over 400 islands within the lakes Uddjaure and Hornaven (N65° 15’ N; 17° 43’ to 66° 99’ N; 17° 55’ E). These islands are not within national reserves, and the land is public and not government protected. We confirm that all national and international rules were complied with during the field work and no specific permissions were required to access the islands. The research did not involve measurements on humans or animals. The soil and plant material collected for this study had negligible effects on broader ecosystem functioning. We have no commercial interests or conflicts of interest in performing this work.

### Study System

The lakes were formed when the retreat of land ice 9,000 years ago left behind granite and moraine boulders to form the foundation of the current islands. All of these islands are of the same geological age and experience the same climatic conditions, having a mean annual precipitation of 750 mm and mean temperature of +13°C in July and -14°C in January. All islands are the same distance from the shore irrespective of island size which prevents confounding effects of erosion, wind and radiation. The forested portions of the islands are not subjected to other major disturbances such as flooding and ice formation, as the level of the lake is always below the level of the lowest forested vegetation. The only extrinsic factor which varies among the islands is wildfire frequency [17–19]. The 30 islands are of varying size (0.02 to 15 ha) with lightning strikes being more frequent on larger islands as they have a larger area to intercept these strikes. The age of major wildfire events was previously determined using ^14^C dating of buried charcoal fragments and through examination of fire scars from the last 250 years [17–20]. These islands collectively form a retrogressive chronosequence, with retrogression increasing with decreasing island size. A description of island properties is given in S1 Table.

### Determining *in situ* CH_4_ fluxes

The *in situ* gas fluxes were measured over one week using a static chamber method during August 2006 (n = 5 per island; chamber volume = 0.0054 m^3^, diameter = 0.2 m). Cylindrical chamber base rings were installed and subsequently removed on the day of measurement. The installation involved gently cutting the base ring into the humus to 5 cm depth. Headspace gas samples, 9 ml, were drawn at 0, 5, 10, 15, 20, 25 and 30 minutes after an opaque chamber lid was sealed onto the base ring. Samples were stored in exetainer vials (Labco, UK) for up to one month prior to analyses. Concentrations of CH_4_ were analysed by gas chromatography, on a PerkinElmer Autosystem XL Gas Chromatograph (GC) with Flame Ionization Detector, and results were calibrated using certified gas standards. CH_4_ was separated isothermally on a 2 m Poropak Q 50–80 mesh packed column at 40°C, with N_2_ as the carrier gas. The mean detectable concentration change was 0.05 ppm CH_4_ which was equivalent to 3 μg CH_4_ m^-2^ hr^-1^ [27]. Where fluxes fell below this detection limit they were still included in the analyses following others [27–29]. Calculations of the rates of exchange of CH_4_ are based on a standard linear regression methodology [30].

### Determining *ex situ* CH_4_ fluxes from intact cores

We measured CH_4_ fluxes from *ex situ* cores from the top 25 cm of soil (or to the bedrock where soil depth was less than 25 cm) in 2006 and then through the entire humus profile to the bedrock (20–85 cm depth) in 2007. In August 2006, four intact cores (11 cm diameter, 25 cm depth) were removed from each island (40 per island size classification) and were weighed on the day of collection. Cores were stored at 12°C in the dark until measurement and were maintained at their field collected moisture content using an artificial rain solution. The artificial rain solution was made up according to a formula which results in rainwater with a pH of 5.6 with a conductivity of 22.4 μS (S4 Table). Cores were grouped into late, mid and early successional stage dependent upon the island from which they were removed (n = 10). Soil CH_4_ fluxes were measured using the static chamber method (volume = 0.0008 m^3^) with sampling at 0, 30, 60, 90, 120 and 150 minutes after headspace closure. In 2007, one full profile core (0.15 m diameter) was collected from each island humus profile to the bedrock in July 2007. A similar static chamber approach (volume = 0.0021 m^3^, diameter = 0.15 m) for measuring soil CH_4_ fluxes, applied under the same measurement conditions as previously described. Sample storage using 3.6 ml exetainers vials, GC analysis and data analysis was as described for the *in situ* CH_4_ sample analyses. Soil moisture was calculated after drying soils in an oven at 105°C until no further moisture loss was recorded.

### *In situ* depth profiles of δ^13^CH_4_ and concentrations of CH_4_, and CO_2_

*In situ* gas samples were collected from within the soil atmosphere in the humus profiles of each island over 4 days in July 2007. Four stainless steel sampling probes (19 gauge, 0.69 mm inner diameter) were inserted into the soil profiles 30 minutes before soil atmosphere samples were collected at 15, 35, 55 and 75 cm depth (dead-space volume = 0.06, 0.13, 0.21, 0.28 ml, respectively). From each probe a 100 ml sample was collected with 90 ml being transferred to a 50 ml Wheaton bottle with butyl rubber stopper and crimp top seals and 9 ml to a 3.6 ml exetainer vial. After collection each butyl rubber stopper and crimp top was sealed with wax. Analyses of the δ^13^C values of CH_4_ were completed within one month at the Natural Environmental Research Council Life Sciences Mass Spectrometry Facility at the Centre for Ecology & Hydrology, Lancaster, using an Isoprime Ltd Tracegas Preconcentrator coupled to an Isoprime Ltd Isoprime isotope ratio mass spectrometer. Samples were initially passed through a Magnesium perchlorate/Carbosorb scrubber trap at 20 ml min^-1^ to eliminate water and CO_2_. The CH_4_ was then oxidized in a combustion furnace using a braided platinum/copper/nichrome furnace wire inside a ceramic furnace tube of 200 mm x 0.4 mm i.d. heated in a furnace at 950°C. A preparation flow rate of 10 psi was required to give a flow rate of 20 ml min^-1^ through the furnace at full operating temperature. Precisions were better than or equal to 0.2 ‰. The δ^13^C of CH_4_ was expressed in per mil and calculated with respect to Pee Dee Belemnite (PDB). Reference CO_2_ was calibrated to National Institute of Standard Technology (NIST) certified reference materials (N.I.S.T. 8559 and 8561) and working CH_4_ standards (Scientific and Technical Gases Ltd).

### Isotopic method to determine the quantity of CH_4_ oxidised

Non-equilibrium or kinetic isotope effects (KIEs) associated with methanotrophy in soils are denoted using the isotope fractionation factor (α_ox_). The KIE represents the different rates of uptake of the CH_4_ isotopologues (^12^CH_4_, ^13^CH_4_), with α_ox_ being the ratio of the rate constants of isotopically light and heavy CH_4_ for both physical and biological processes associated with methanotrophy in soil [24–25]. The resultant isotopic fractionation factor for CH_4_ oxidation (α_ox_) is known to vary across boreal soils [24], therefore it was necessary to determine α_ox_ for each island. A straightforward and effective method of determining α_ox_ is by using the ‘Top-to-Bottom’ δ^13^C-CH_4_ approach [24–25]. This allows the determination of the fraction of atmospheric CH_4_ as it passes through soil layers. It involves calculating the difference in δ^13^C-CH_4_ values from atmospheric CH_4_ samples and samples taken from the soil atmosphere at depth. This difference is then added to 19.7 ‰ to take account of fractionation by gas diffusion through the soil due to the higher translational velocity of ^12^CH_4_ versus ^13^CH_4_ in air filled pores spaces. Diffusion of CH_4_ into the soil tends to result in isotopic depletion as ^12^CH_4_ diffuses more quickly along its gradient than ^13^CH_4_ causing ^12^CH_4_ to travel further in soil pore space. During the process of CH_4_ oxidation the remaining CH_4_ becomes heavier as ^12^CH_4_ is more readily oxidised by methanotrophs. The calculated isotope fractionation factors (α_ox_) for this work are given in S4 Table.

To determine the fraction of CH_4_ oxidised by boreal forest soils, a mass balance model was used that accounted for the fractionation effects of both biological oxidation and transport of CH_4_ from the atmosphere. The model is adapted from previous work that estimated CH_4_ oxidation in landfill [31] cover soils, but has since been used in natural systems to investigate CH_4_ cycling in humid tropical forests [32]. Eq 1 describes the model:

$$Fo=(\delta_{soil}-\delta_{atmos})/[(\alpha_{ox}-\alpha_{trans})\times 1000]$$ Eq 1

where F_o_ represents the fraction of CH_4_ oxidised. To calculate the percentage of CH_4_ oxidised, F_o_ is multiplied by 100. δ_soil_ and δ_atmos_ represent the isotopic composition (‰) of CH_4_ at soil depths and atmosphere respectively. α_trans_ is the isotopic fractionation associated with gas transport. There is no method to directly calculate α_trans_. Other studies have assumed movement of CH_4_ by advective transport [31] which involves no isotopic fractionation of CH_4_ giving a α_trans_ value of 1. Using this value can result in an underestimation of CH_4_ oxidation. As in this study, it was not possible to calculate α_trans_, F_o_ was calculated using α_trans_ values of 1.0195, which is the diffusion fractionation factor reported for temperate forest soils [33].

### Ecosystem properties

Using our 2006 collected soil cores, we measured soil pH, % carbon and % nitrogen. Soil pH was determined in a soil:water ratio of 1:3 using the Hanna pH 210 Microprocessor pH Meter. The percentage of soil C and N was analysed by oxidative combustion, followed by thermal conductivity detection using an Elementar Vario EL analyser (Elementar Analysensysteme GmbH, Hanau, Germany). In July 2007 the humus depth of each island was assessed by taking ten depth measurements along a transect across approximately the centre of each island. Depths were sampled at equal intervals by inserting a metal rod to the bedrock. Additional existing information on vegetation mass, shrub productivity, tree productivity and humus mass are as reported previously [17–20].

### Statistical Analyses

All analyses were conducted with the R statistical program (R Development Core Team, 2011). Simple regression analyses were used to model the relationship between response variables and predictors (successional age of system from ^14^C dating, humus development). Differences in island soil moisture content, *in situ* and *ex situ* CH_4_ fluxes between successional stages (Early, Mid and Late) were tested with the *glm* function. This same approach was applied when testing for differences between the quantity of CH_4_ oxidised for successional stages using the stable isotope approach. A constant value, equal to lowest datapoint + 1, was added to each datapoint to ensure all data were non-negative and a quasi-likelihood function with a log link and constant variance parameter used to account for the non-normal distribution. Differences in other CH_4_ measures (CH_4_ concentration, δ^13^C of CH_4_ and % CH_4_ oxidised) across successional stages and soil depths were tested with a linear mixed effect model using the *lme* function in the *nlme* package. Island was included as a random term to account for dependencies between measures made on the same core. Diagnostic plots of residuals versus fitted values were inspected to ensure there was no bias or heteroscedasticity in model residuals.

## Supporting Information

S1 Table Changes in island properties for each successional class across the island size gradient. (DOCX)

S2 Table Mean CH_4_ concentration (ppm) in soil air of *ex situ* cores at stratified depths. (DOCX)

S3 Table Statistical analyses for the effects of, and interactions between, depth and successional stage on *ex situ* profile CH_4_ concentrations. (DOCX)

S4 Table Kinetic Isotope Effect (KIE) for soil methanotrophy on early, mid and late succession islands. (DOCX)

S5 Table Artificial Rain Solution. (DOCX)
