# Supplementary material for: Soil Methane Sink Capacity Response to a Long-Term Wildfire Chronosequence in Northern Sweden
Source: PLoS One. 2015 Sep 15;10(9):e0129892. doi: 10.1371/journal.pone.0129892 (PMC4570772; doi:10.1371/journal.pone.0129892)
Supplement: S1 Table — (DOCX) [file pone.0129892.s001.docx]

**S1 Table. Changes in island properties for each successional class across the island size gradient.** Values in brackets are standard errors of the mean. Values followed by the same letter in subscript indicate no significant difference at P < 0.05. (2,27 d.f.; n=10 for each size class).

|  |  | | | | |  | |  |
| --- | --- | --- | --- | --- | --- | --- | --- | --- |
| **Island Properties** | | **Large Island**  **(>1.0 ha)** | **Medium Island**  **(0.1-1.0 ha)** | | **Small Island**  **(<0.1 ha)** | | **F (P)** | |
|  | |  | |  |  | |  | |
| **Humus depth (cm)** | | 28.4 (2.1)^a^ | | 42.4 (4.6)^b^ | 65.9 (4.6)^c^ | | 22.66 (0.000) | |
| **Bulk Density (g/cm^3^)** | | 0.15 (0.02)^a^ | | 0.11 (0.01)^ab^ | 0.08 (0.00)^b^ | | 0.53 (0.02) | |
| **pH** | | 3.9 (0.1)^a^ | | 3.7 (0.0)^a^ | 3.8 (0.1)^a^ | | 0.94 (0.404) | |
| **%C** | | 30.7(5.8)^a^ | | 42.0 (5.0)^a^ | 53.5 (0.3)^b^ | | 6.67 (0.004) | |
| **%N** | | 0.7 (0.1)^a^ | | 1.2 (0.2)^b^ | 1.7 (0.1)^c^ | | 13.56 (0.000) | |
| **%P** | | 0.06 (0.01)^a^ | | 0.07 (0.00)^a^ | 0.07 (0.00)^a^ | | 1.03 (0.371) | |
| **C:N** | | 41.6 (1.3)^a^ | | 36.6 (1.7)^b^ | 32.7 (1.2)^b^ | | 9.62 (0.001) | |
| **N:P** | | 12.1 (2.1)^a^ | | 16.7 (2.3)^a^ | 25.3 (1.6)^b^ | | 11.11 (0.000) | |
| **C:P** | | 516.6 (99.9)^a^ | | 583.5 (66.4)^a^ | 813.0 (33.6)^b^ | | 4.67 (0.018) | |
