# Supplementary material for: Soil Methane Sink Capacity Response to a Long-Term Wildfire Chronosequence in Northern Sweden
Source: PLoS One. 2015 Sep 15;10(9):e0129892. doi: 10.1371/journal.pone.0129892 (PMC4570772; doi:10.1371/journal.pone.0129892)
Supplement: S2 Table — (DOCX) [file pone.0129892.s002.docx]

**S2 Table.** **Mean CH_4_ concentration (ppm) in soil air of *ex situ* cores at stratified depths.** Values in brackets are standard errors of the mean (n=10). Statistical analyses are shown in Table S3.

|  | **Successional Stage** | | |
| --- | --- | --- | --- |
| **Depth (cm)** | **Early** | **Mid** | **Late** |
| **5** | 1.92 (0.03) | 1.86 (0.02) | 1.78 (0.03) |
| **15** | 1.84 (0.05) | 1.71 (0.04) | 1.63 (0.07) |
| **25** | 1.82 (0.10) | 1.49 (0.06) | 1.42 (0.11) |
| **35** | 1.81 (0.13) | 1.43 (0.12) | 1.35 (0.13) |
| **45** | - | 1.03 (0.06) | 1.29 (0.10) |
| **55** | - | 1.87 (0.00) | 1.30 (0.14) |
| **65** | - | - | 1.57 (0.16) |
| **75** | - | - | 1.79 (0.05) |
