# Supplementary material for: Soil Methane Sink Capacity Response to a Long-Term Wildfire Chronosequence in Northern Sweden
Source: PLoS One. 2015 Sep 15;10(9):e0129892. doi: 10.1371/journal.pone.0129892 (PMC4570772; doi:10.1371/journal.pone.0129892)
Supplement: S3 Table — (DOCX) [file pone.0129892.s003.docx]

**S3 Table.** **Statistical analyses for the effects of, and interactions between, depth and successional stage on *ex situ* profile CH_4_ concentrations.**

| **CH_4_ concentration** | | F | | P | |  |
| --- | --- | --- | --- | --- | --- | --- |
| Stage (S) | | 13.046 | | <0.0001 | |  |
| Sample Depth (SD) | | 23.046 | | 0.0001 | |  |
| Stage x Sample Depth (S x SD) | | 3.142 | | <0.0001 | |  |
|  |  | |  | |  | |
|  |  | |  | |  | |
|  |  | |  | |  | |
|  |  | |  | |  | |
