# Supplementary material for: Soil Methane Sink Capacity Response to a Long-Term Wildfire Chronosequence in Northern Sweden
Source: PLoS One. 2015 Sep 15;10(9):e0129892. doi: 10.1371/journal.pone.0129892 (PMC4570772; doi:10.1371/journal.pone.0129892)
Supplement: S4 Table — (DOCX) [file pone.0129892.s004.docx]

**Table S4.** **Kinetic Isotope Effect (KIE) for soil methanotrophy on early, mid and late succession islands.**

| **Successional Stage** | **0-15 cm** | **n** | **0-35 cm** | **n** | **0-55 cm** | **n** | **0-75 cm** | **n** |
| --- | --- | --- | --- | --- | --- | --- | --- | --- |
| **Early** | 1.0208  (0.0004) | 10 | 1.0215  (0.0004) | 7 | nd | nd | nd | 0 |
| **Mid** | 1.0206  (0.0005) | 10 | 1.0223  (0.0006) | 9 | 1.0211  (0.0008) | 6 | nd | 0 |
| **Late** | 1.0211  (0.0005) | 10 | 1.0223  (0.0010) | 10 | 1.0237  (0.0006) | 9 | 1.0219  (0.0011) | 4 |

Values in brackets are standard errors of the mean. ‘nd’ indicates where samples could not be obtained due to a shallow humus depth. The number of depths sampled is given by n.
