# Supplementary material for: Soil Methane Sink Capacity Response to a Long-Term Wildfire Chronosequence in Northern Sweden
Source: PLoS One. 2015 Sep 15;10(9):e0129892. doi: 10.1371/journal.pone.0129892 (PMC4570772; doi:10.1371/journal.pone.0129892)
Supplement: S5 Table — (DOCX) [file pone.0129892.s005.docx]

## Table S5. Artificial Rain Solution. Contents added to 2 L deionised water.

| **Formula** | **Chemical** | **Weight (g)** |
| --- | --- | --- |
| NH_4_NO_3_ | Ammonium nitrate | 6.9 |
| NH_4_CL | Ammonium chloride | 2.4 |
| KCL | Potassium chloride | 2.0 |
| MgSO_4_.7H_2_O | Magnesium sulphate | 16.3 |
| CACl_2_.6H_2_O | Calcium chloride hexahydrate | 13.8 |
| NaCL | Sodium chloride | 20.6 |
| Na_2_SO_4_ | Sodium sulphate anhydrous | 3.2 |
